# Safety and effectiveness of new embolization microspheres SCBRM for intermediate-stage hepatocellular carcinoma: A feasibility study

**DOI:** 10.17305/bjbms.2020.4770

**Published:** 2021-06

**Authors:** Yi-Sheng Liu, Xi-Zhang Lin, Chiung-Yu Chen, Yen-Cheng Chiu, Jui-Wen Kang, Hung-Wen Tsai, Hui-Yu Hung, Chi-Ming Ho, Ming-Ching Ou

**Affiliations:** 1 Department of Medical Imaging, National Cheng Kung University Hospital, College of Medicine, National Cheng Kung University, Tainan, Taiwan; 2 Department of Internal Medicine, National Cheng Kung University Hospital, College of Medicine, National Cheng Kung University, Tainan, Taiwan; 3 Department of Pathology, National Cheng Kung University Hospital, College of Medicine, National Cheng Kung University, Tainan, Taiwan

**Keywords:** Biodegradable, hepatocellular carcinoma, transcatheter arterial chemoembolization, microspheres, safety, efficacy

## Abstract

Transarterial chemoembolization (TACE) is, currently, the recommended treatment for hepatocellular carcinoma (HCC). However, long-term chemoembolization triggers the inflammatory response and may lead to postembolization syndrome (PES). Although several types of degradable microspheres have been developed to reduce drug toxicity and PES incidence, the clinical outcomes remain unsatisfactory. Previously, we have developed a new type of spherical, calibrated, biodegradable, radiopaque microspheres (SCBRM) and demonstrated their safety and efficacy in a pig model. Thus, the goal of this feasibility study was to determine the clinical safety and efficacy of the new SCBRM in intermediate-stage HCC patients. In this study, 12 intermediate-stage HCC patients underwent TACE using SCBRM with a calibrated size of 100–250 μm. The disease control rates at 1 month and 3 months after TACE-SCBRM treatment were 100% and 75.0%, respectively. The objective response rates at 1 month and 3 months after treatment were 66.7% and 58.3%, respectively. Very few adverse events were observed with one patient developing nausea. One day after the treatment, alanine aminotransferase, alanine aminotransferase, and total bilirubin levels were slightly elevated in the patients, but all returned to baseline on day 7. The median and mean overall survival times were 33 months (interquartile range, 12.8–42.0) and 29.2 ± 14.3 months, respectively. The 1-year and 2-year survival rates were 91.7% and 58.3%, respectively. In conclusion, TACE with the new SCBRM microspheres is clinically safe and effective, and it represents a promising approach in the management of intermediate-stage HCC.

## INTRODUCTION

Hepatocellular carcinoma (HCC) is the sixth most common tumorous cancer and the third most deadly cancer worldwide [1,2]. It accounts for more than 80% of primary liver cancers worldwide [2]. Although advanced imaging methods and long-term monitoring of patients with chronic liver diseases are used to screen for early-stage liver cancer, advanced stage liver cancer remains resistant to cure [3,4]. Current curative strategies for early-stage HCC include orthotopic liver transplantation, liver resection, and radiofrequency ablation (RFA) [5]. However, many patients with HCC are diagnosed at advanced stage and cannot be treated with these options. Although liver transplantation can cure underlying liver disease and advanced cirrhosis, organ shortage and the possibility of lifelong immunosuppression are major limitations to its use [5,6]. At the Barcelona meeting in 2000, experts and scholars recommended using non-invasive methods and combined laboratory data as diagnostic criteria for liver cancer [7]. Although there are some discrepancies in HCC treatment guideline between different countries, the Barcelona Clinic Liver Cancer (BCLC) Guidelines [8], the Japan Society of Hepatology Consensus-Based Clinical Practice Guidelines [9], and the Hong Kong Liver Cancer prognostic classification scheme [10] all recommend transarterial chemoembolization (TACE) as the gold standard for the treatment of intermediate-stage HCC patients.

TACE has been found to be as effective as hepatic resection and RFA in patients with small single-nodule HCC. Furthermore, in early-stage HCC patients, the overall survival (OS) with TACE was similar to that of hepatic resection or RFA [11,12]. Conventional TACE (cTACE) injects an emulsion of chemotherapeutic agents (e.g., doxorubicin) and Lipiodol^®^ into the tumor-feeding branches of the hepatic artery, followed by injection of an embolic agent (e.g., Embosphere^®^). In addition to providing high concentrations of chemotherapeutic drug for tumor tissues, this procedure also blocks the blood vessels feeding the tumors, preventing their access to oxygen and nutrients. The embolic materials not only cause tumor ischemia and necrosis, but also slow down the washout of the injected therapeutic drug. However, tissue ischemia caused by long-term chemoembolization and the consequent inflammatory response and release of cytokines often lead to postembolization syndromes (PES). Typical symptoms include fever, nausea, vomiting, and right upper quadrant pain; severe complications include cholecystitis, liver failure, liver abscess, and intraperitoneal hemorrhage [13-15]. In addition, the common TACE-induced adverse reactions include abdominal or flank pain, fatigue, weakness or sleepiness, nausea, dizziness, decreased appetite or anorexia, vomiting, constipation, and insomnia. The typical embolic agents used in cTACE treatment, iodized oil (Lipiodol^®^) and polyvinyl alcohol, can cause permanent embolization of the hepatic artery [16]. In addition to the increased risk of PES, the use of permanent embolic agents has certain limitations. For example, prolonged ischemia may increase the expression of vascular endothelial growth factor, thereby promoting the growth of new tumor vessels [17]. Thus, degradable microspheres (e.g., EmboCept^®^, PharmaCept, Berlin, Germany; Spherex^®^, Magle Life Science, Lund, Sweden) are also used clinically for chemoembolization to temporarily block arterial blood vessels to achieve therapeutic effects and reduce both drug toxicity and the incidence of PES [18]. The half-life of EmboCept^®^ and Spherex^®^ is about 35 and 15 minutes, respectively, providing transient occlusion of small arteries. EmboCept^®^ is currently available in one size (50 mm).

Recently, we developed a new type of spherical, calibrated, biodegradable, radiopaque microspheres (SCBRM) with a size of 100–250 μm. SCBRM showed temporary arterial embolization of the liver and spleen of pigs, and its effect and safety were equivalent to or better than those of both Gelfoam^®^ and Embosphere^®^ [19]. Thus, the aim of this feasibility study was to evaluate the safety and efficacy of SCBRM in HCC patients with BCLC-B stage cancer.

## MATERIALS AND METHODS

### Patient selection and ethics

This feasibility study was conducted from March 2016 to February 2017 in accordance with the Declaration of Helsinki, and the protocol was approved by the Institutional Review Board of the hospital (No. #B-BR-104-046). All patients signed written informed consent to participate. The inclusion criteria were patients older than 18 years of age and pathologically confirmed as having BCLC-B stage HCC (intermediate-stage HCC) and a survival time >3 months. BCLC-B stage HCC was classified in accordance with the BCLC grading standards: a tumor size of 3–6 cm, liver function <9 on the Child-Pugh scale (Child-Pugh A or B), and a difficult to remove tumor surgically, not suitable for surgery, or the patient was unwilling to have surgery. In addition, patients diagnosed with liver cancer met one of the following criteria: 1) liver cancer, confirmed histopathologically by the clinical physician; 2) high-risk liver cancer due to viral hepatitis or cirrhosis, confirmed by at least two imaging examinations (ultrasound, computed tomography [CT] scan, magnetic resonance imaging [MRI], or angiography); or 3) tumor progression at the same location of high-risk liver cancer caused by viral hepatitis or cirrhosis, confirmed by two consecutive follow-up visits. The exclusion criteria were: 1) portal vein embolism or metastasis outside of the liver; 2) other malignant tumors; 3) decompensated liver cirrhosis (total bilirubin [T-bil] >2, prothrombin time >3 seconds, aspartate transaminase [AST] >500 U/L, alanine aminotransferase [ALT] >500 U/L, refractory ascites, active bleeding, hepatic coma, or infection); 4) poor kidney function (creatinine [Cr] >2.0 mg/dL and estimated glomerular filtration rate [eGFR] <50 ml/minutes/1.73 m^2^); 5) allergic to iodine-containing contrast agents or drugs that must be injected; 6) other major organ failure (i.e., heart, lung, or kidney); 7) decreased leukocytes (white blood cells [WBC] <3000/mm^3^, absolute neutrophil count <1500/mm^3^) or severe thrombocytopenia (platelet count <50,000/μL); 8) tumors that cannot be imaged or tracked with ultrasound or CT; 9) pregnant woman; and 10) blood vessels too complex or too small to be embolized.

### Manufacture of new SCBRM

The new SCBRM were constructed as previously reported [19]. SCBRM were constructed using water insoluble and biodegradable excipients, including cetyl alcohol, cholesterol, glycol monostearate, Lipiodol^®^, polycaprolactone, and stearyl acid. Calibrated SCBRM microspheres were produced using the atomization technique and high frequency resonated technique. The size of the microspheres was further confirmed by using a scanning electron microscope. In this study, SCBRM with a size of 100–250 mm were used to embolize the intrahepatic artery.

### Procedure of SCBRM-based TACE

TACE with emulsion-based formulations using doxorubicin and Lipiodol^®^ was performed. After disinfection and draping, the physician injected a local anesthetic with 2% lidocaine (5–10 ml) at the planned puncture site in the left or right groin using the Seldinger technique. The physician then made a puncture nearby, and a 4F angiocatheter (4Fr. Yashiro catheter, Terumo Corporation, Tokyo, Japan) was inserted into the femoral artery. After the catheter reached the hepatic artery, diagnostic angiography was performed to clarify the location of the lesion and the blood vessels supplying the tumors. A 2.7F microcatheter (Progreat Microcatheter System, Terumo, Japan) was inserted into the branch of the artery supplying the tumors, and an emulsion of Lipiodol^®^ and doxorubicin was injected (1 mL Lipiodol^®^ to 5 mg doxorubicin). SCBRM microspheres were then injected into the targeted blood vessel until either the blood supply was cut off or the blood flow was embolized to the second and third branches of the hepatic artery before blood backflow occurred. Angiography was performed again to confirm that the embolization was complete. The catheter was then removed and an arterial hemostat was used to stop the bleeding at the wound site on the femoral artery to reduce the risk of complications due to local bleeding. In this study, all patients received one SCMRM-based TACE. The patients were then discharged within 3–7 days based on their clinical status.

### Statistical analysis

All data were statistically analyzed using SAS software version 9.4 (SAS Institute Inc., Cary, NC, USA). Frequency and percentage were summarized for categorical variables. Numeric variables were presented as median and interquartile range (IQR) or presented as mean ± standard deviation (SD). Wilcoxon signed-rank test was used to compare the biochemistry values at 1 day and 1 week after SCBRM-TACE treatment. A two-tailed *p* < 0.05 was considered statistically significant.

## RESULTS

### Patient characteristics

The demographic characteristics of the 12 patients enrolled in this study are presented in Table 1. The mean age of the HCC patients was 71.3 ± 8.1 years, and 58.3% of patients were male. Most patients had hepatitis virus infection (91.7%), 41.7% had hepatitis B virus, and 50.0% had hepatitis C virus. Most patients (75.0%) had Child-Pugh A cirrhosis. All patients were classified as having BCLC stage B disease. Most patients (75%) had ≤3 tumor nodules. The average tumor size was 3.6 ± 0.4 cm (median 3.5, range 3.1–4.2 cm). The tumors were located in the left, right, and bilateral lobes of 3, 7, and 2 patients, respectively. The proportions of these patients who previously received TACE, RFA, percutaneous ethanol injection (PEI), and hepatic resection were 75%, 58.3%, 25%, and 16.7%, respectively.

**TABLE 1:**
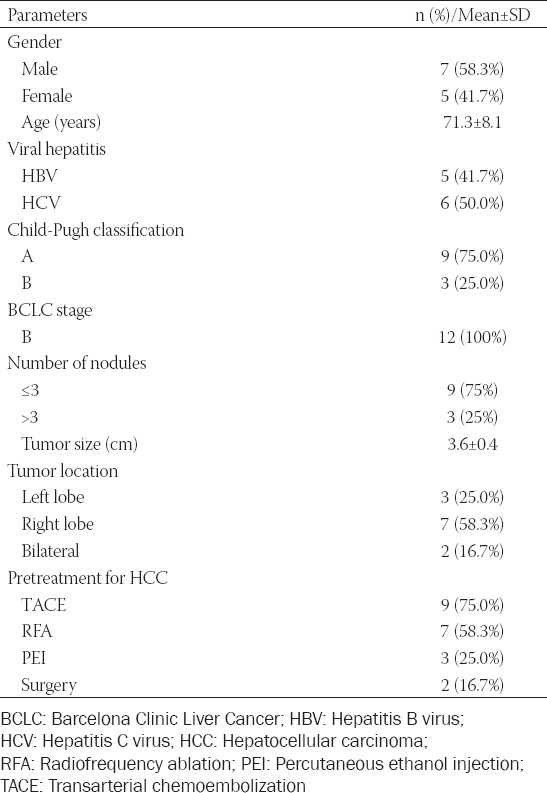
Baseline demographic and general characteristics of BCLC-B HCC patients

### Safety

The biochemical measurements and adverse events of HCC patients after TACE with SCBRM were recorded. Compared with baseline, the levels of AST and ALT were elevated on the 1^st^ day after treatment (Table 2, 54.42 ± 23.64 U/L vs. 105.92 ± 107 U/L; 35.5 ± 23.98 U/L vs. 60.25 ± 47.84 U/L, respectively, *p* > 0.05). T-bil was significantly increased from 0.75 ± 0.39 mg/dL to 1.38 ± 0.85 mg/dL (*p* < 0.05). Prothrombin time was slightly increased from 11.6 ± 0.91 seconds to 12.56 ± 1.22 seconds (*p* < 0.05), while albumin level was slightly decreased from 4.03 ± 0.74 g/dL to 3.97 ± 0.54 g/dL (*p* > 0.05). There was no significant difference in the change of Cr level (0.95 ± 0.26 mg/dL vs. 0.87 ± 0.25 mg/dL; *p* > 0.05). On the other hand, blood urea nitrogen and the WBC count remained within normal ranges 1 day after TACE treatment (21.17 ± 7.91 mg/dL vs. 15.75 ± 3.72 mg/dL; 5.62 ± 1.57 10^3^/mm^3^ vs. 6.98 ± 1.32 10^3^/mm^3^, respectively; *p* < 0.05). There was no significant difference in α-fetoprotein levels before and 1 month after treatment (*p* > 0.05). All these levels returned to baseline levels 7 days after treatment. Table 3 shows the adverse events within 3 months after SCBRM embolization. One patient had nausea symptoms after treatment and no other adverse events were observed.

**TABLE 2:**
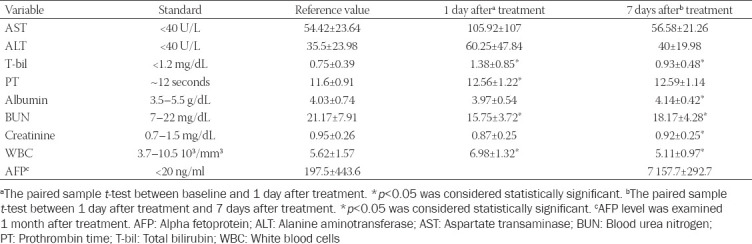
Biochemistry values at different time points before and after TACE treatment

**TABLE 3:**
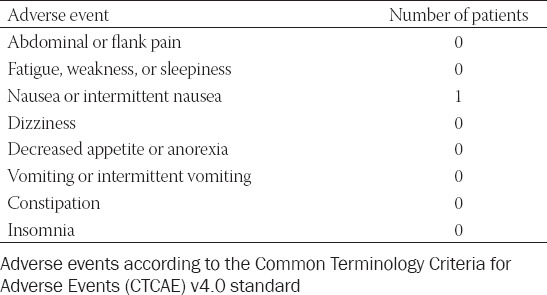
Adverse events within 3 months after treatment

### Efficacy

Follow-up CT and MRI examinations were used to analyze the tumor response at 1 and 3 months after SCBRM embolization (Table 4). After 1 month of treatment, the number of patients who achieved complete response (CR), partial response (PR), stable disease (SD), and progressive disease (PD) were 3 (25%), 5 (41.7%), 4 (33.3%), and 0 (0%), respectively. After 3 months of treatment, these numbers were 3 (25%), 4 (33.3%), 2 (16.7%), and 2 (16.7%), respectively. The objective response rates (ORR) at 1 and 3 months after treatment were 66.7% and 58.3%, respectively, while the disease control rates (DCR) at 1 and 3 months after treatment were 100% and 75.0%, respectively.

**TABLE 4:**
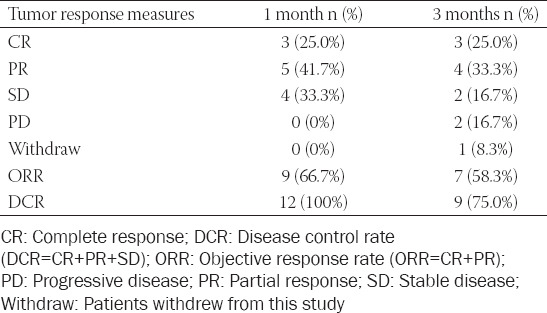
Treatment response at different time points

**FIGURE 1:**
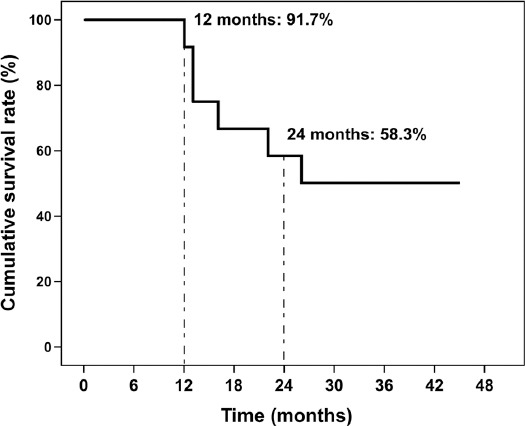
Kaplan–Meier survival curve of Barcelona clinic liver cancer (BCLC)-B hepatocellular carcinoma (HCC) patients after transarterial chemoembolization (TACE)-spherical, calibrated, biodegradable, radiopaque microspheres (SCBRM) treatment.

**FIGURE 2:**
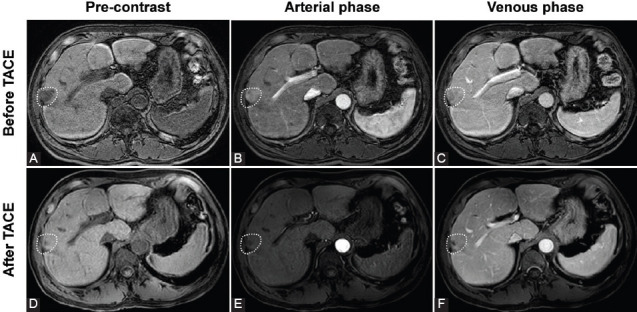
Magnetic resonance images of a successful spherical, calibrated, biodegradable, radiopaque microspheres (SCBRM)-based TACE treatment in a representative patient. (A-C) Baseline imaging of the patient. (A) Pre-contrast T1-weighted image showed the tumor located at the right hepatic lobe with a size of about 2.6 cm (white circle). (B) Arterial phase T1-weighted image showed arterial enhancement. (C) Venous phase T1-weighted image showed the washout. (D-F) After the SCBRM-based transarterial chemoembolization (TACE) treatment. (D) Pre-contrast T1-weight image showed tumor shrinkage. (E) Arterial phase T1-weighted image showed no arterial enhancement. (F) Venous phase T1-weighted image showed no washout enhancement.

Figure 1 depicts the Kaplan–Meier survival curve of patients following TACE using SCBRM. The median and mean OS times were 33 months (IQR, 12.8–42.0) and 29.2 ± 14.3 months, respectively. According to the Kaplan–Meier survival curve, the 1-year and 2-year survival rates were 91.7% and 58.3%, respectively. A successful case is shown in Figure 2. A patient had a tumor at the right hepatic lobe of about 2.6 cm (white circle). After SCBRM-based TACE treatment, T1-weighted MRI showed tumor shrinkage.

## DISCUSSION

Prolonged embolization may not only promote tumor angiogenesis but also increase the risk of PES. Thus, various biodegradable embolic materials have been developed. In our previous study, the new biodegradable microspheres SCBRM that we developed showed promising safety and efficacy in pigs [19]. In the present feasibility study, we further explored the safety and efficacy of SCBRM in BCLC-B HCC patients. The median survival of patients receiving cTACE using SCBRM as an embolic agent was 33 months, and the 1-year and 2-year survival rates were 91.7% and 58.3%, respectively. The 1-month and 3-month ORR were 66.7% (25% CR and 41.7% PR) and 58.3% (25% CR and 33.3% PR), respectively. Of note, no adverse events were observed, apart from nausea in one patient. Liver and kidney functions were slightly changed 1 day after TACE-SCBRM treatment, but all values returned to baseline 7 days after treatment. Therefore, the results of our study suggest that our novel SCBRMs are safe and effective for TACE.

In addition to the common biodegradable embolic agent of Gelfoam particles, recently-marketed degradable starch microspheres (EmboCept^®^S) have shown acceptable DCR (83.3%) at 1-month follow-up and a 1-year OS rate of 66.6% [20]. All 6 enrolled patients were male and at BCLC-C stage. In the study of Iezzi et al., 18 BCLC-B HCC patients and 22 BCLC-C patients received TACE with degradable starch microspheres [21]. The results showed 52.5% DCR, with a median OS of 11.3 months at 1-year follow-up. The BCLC-B HCC patients had a 1-year OS of 64.2%, a 2-year OS of 30.4%, and a median OS of 11.7 months. In a recent study, Gross and Albrecht treated 26 BCLC-B, 8 BCLC-C, and 1 BCLC-D HCC patients with degradable starch microspheres and TACE and showed 49% ORR, 83% DCR, and median survival of 19 months [22]. The BCLC-B HCC patients in that study had a 7.6% CR and a 46.2% PR, with an ORR of 53.8%. In our study, all patients were BCLC-B HCC patients. Moreover, SCBRM showed a better tumor response than in the previous studies, with 66.7% ORR and 100% DCR at 1 month after treatment, and 58.3% ORR and 75.0% DCR at 3 months after treatment. Furthermore, compared with the above reports using EmboCept^®^, the mean OS of our patients was better (up to 33 months) and the 1-year survival rate was 91.7%.

Compared with cTACE-induced ischemia and strong cytotoxicity, doxorubicin-loaded drug eluting beads (DEB-TACE) have a controlled release of cytotoxic drugs and, therefore, have higher safety, better tumor response, and a better survival rate [23-25]. Liu et al. showed that doxorubicin-loaded DEB-TACE has greater long-term benefits than cTACE in HCC patients who have not received TACE before [26]. In general, the ORR of DEB-TACE in HCC patients ranges 39–76%. In the clinical study by Rahman et al., the ORR of HCC patients after DEB-TACE treatment was 39%, and the median survival was 8.3 ± 2.0 months [27]. Another study by Liu et al. in Korea reported an ORR of 60.4% and a DCR of 64.2%, with minor adverse events also observed (5.7%) [28]. Although a recent study by Zhou et al. showed a better ORR (75.8%), these DEB-TACE patients had a higher rate of adverse events, such as pain in 96.0% of patients, fever in 76.8% of patients, and vomiting in 16.1% of patients [29]. Compared with the above DEB-TACE studies, TACE with SCBRM has a relatively good efficacy, with an ORR of 58.3% and a DCR of 75.0%. In addition, SCBRM were also relatively safe, with one patient experiencing nausea, indicating the certain clinical application value of SCBRM.

The low incidence of PES using SCBRM may be due to the biodegradable characteristics of the microspheres. Several studies have suggested that temporary embolization can reduce PES [22,30,31]. For example, Pieper et al. observed no complications in a swine model when degradable starch microspheres were used for temporary arterial embolization of liver parenchyma [18]. Gross and Albrecht also reported only grade 1 adverse events in 37 HCC patients treated with TACE using degradable starch microspheres [22]. For HCC patients refusing or ineligible for sorafenib, degradable starch microspheres were also associated with a low rate of minor complications (15%) [21]. No major complications or treatment-related deaths were observed. The above studies are consistent with our results that TACE with biodegradable microspheres can reduce the occurrence of PES.

Recent studies have shown that patients with HCC receiving smaller microspheres showed better tumor response, better survival outcomes, fewer adverse events, and fewer patient complications [32,33]. However, using microspheres that are too small may lead to worse results. In a comparison of 70–150 µm and 100–300 µm DEB particles [34], the HCC patients receiving TACE with 70–150 µm DEB particles showed 17% CR, 28% PR, 33% SD, and 22% PD, with an ORR of 61%. In contrast, for HCC patients receiving 100–300 µm TACE-DEB, these were 14% CR, 50% PR, 7% SD, and 29% PD, with an ORR of 64%. Therefore, in this feasibility study, we used SCBRM microspheres of 100–250 µm, and the results did show good efficacy and safety.

The present study has several limitations. First, this study evaluated the safety and efficacy of the new SCBRM with one calibrated size of 100–250 μm. Although the safety and effectiveness were promising, the effects of different SCBRM sizes and the differences between SCBRM and other commercial microspheres remain unclear. Second, this study examined safety and efficacy in a small number of BCLC-B HCC patients. Despite the promising results of our study, further prospective studies, optimally of randomized controlled design, should recruit more participants at different BCLC stages and fully compare different microsphere sizes of SCBRM, as well as compare SCBRM treatment with treatment utilizing other available microspheres. Third, since SCBRM is a drug-loadable microsphere, the results for SCBRM in DEB-TACE remain unclear in this study. We are currently conducting another clinical trial to examine the effectiveness of SCBRM in DEB-TACE.

## CONCLUSION

This feasibility study provides some evidence of the efficacy and safety of a new type of SCBRM with a size of 100–250 μm for the treatment of patients with intermediate-stage HCC. Although this study shows promising results, these should be verified in further clinical trials.
